# Ectopic Pregnancy in a Cesarean Section Scar: Successful Management Using Vacuum Aspiration under Laparoscopic Supervision—Mini Review of Current Literature

**DOI:** 10.1155/2016/7460687

**Published:** 2016-11-24

**Authors:** Mustafa Koplay, Nasuh Utku Dogan, Mesut Sivri, Hasan Erdogan, Selen Dogan, Cetin Celik

**Affiliations:** ^1^Department of Radiology, Medical Faculty, Selcuk University, Konya, Turkey; ^2^Department of Obstetrics and Gynecology, Medical Faculty, Akdeniz University, Antalya, Turkey; ^3^Department of Obstetrics and Gynecology, Medical Faculty, Selcuk University, Konya, Turkey

## Abstract

A cesarean scar ectopic pregnancy (CSEP) is a fairly uncommon presentation wherein the conceptus is implanted deep in the myometrium and at the exact scar site of the previous cesarean section. There are various CSEP management options that range from medical treatment to surgical interventions such as dilatation and curettage, laparoscopic excision, resection by laparotomy, or, sometimes, a combination of these modalities. Establishing a diagnosis of CSEP can be challenging. Given the relatively rare incidence of CSEP, its management is controversial and current standards of therapy have been derived from data obtained from a limited number of patients. Herein, we present transvaginal ultrasonography (TVUS) imaging findings and management strategies used in a case of CSEP along with the short review of current literature.

## 1. Introduction

A cesarean scar ectopic pregnancy (CSEP) is a fairly uncommon presentation wherein the conceptus is implanted deep in the myometrium and at the exact scar site of the previous cesarean section [1]. Diagnosis and appropriate and timely management of CSEP are essential because if left untreated, it may lead to serious complications such as uterine rupture, hemorrhage, hypovolemic shock, disseminated intravascular coagulation, and even maternal death [2]. The gold standard for diagnosing CSEP is transvaginal ultrasonography (TVUS). There are various CSEP management options that range from medical treatment to surgical interventions such as dilatation and curettage, laparoscopic excision, resection by laparotomy, or, sometimes, a combination of these modalities. Herein, we present TVUS imaging findings and management strategies used in a case of CSEP along with the short review of current literature in order to show that, even in complicated clinical scenarios, minimally invasive surgical techniques prove to be a valuable approach.

## 2. Case

**Figure 1:**
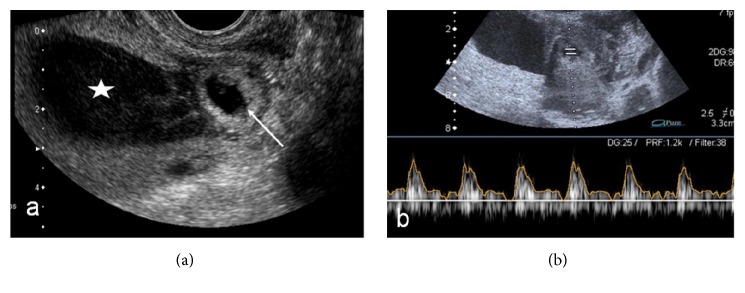
(a) A TVUS examination showing the gestational sac (arrow) near isthmus, close to previous cesarean section scar, and hemorrhagic fluid in endometrial cavity (star). (b) Spectral Doppler US showing the fetal pole and fetal heart rate in gestational sac.

A 25-year-old gravida 3 para 2 woman at 7 weeks of pregnancy was admitted to our unit with vaginal bleeding. Siblings from both previous pregnancies had been delivered by cesarean section. Besides these two surgeries, her medical history was unremarkable. Physical examination showed no abdominal tenderness or rebound. Speculum examination revealed a normal cervix with minimal bleeding and without any cervical dilatation. Her blood pressure (110/70 mmHg) and pulse rate (80 beats/min) were within normal limits; hemoglobin was 13.2 g/dL and hCG was 38067 mIU/mL. A TVUS (Toshiba Aplio, Tokyo, Japan) performed at admission showed that there was no intrauterine gestational sac or fetal pole and that the intrauterine cavity was filled with hemorrhagic fluid. However, a gestational sac of dimensions 36 × 42 mm diameter and a viable fetus with crown to lump length of 5.3 mm were visualized near the isthmus and at the exact location of the scar from the previous cesarean section (Figure 1(a)). A color Doppler US demonstrated proliferative growth of the peritrophoblastic vessels around the gestational sac, and a spectral Doppler US showed the fetal heart activity (Figure 1(b)). There was no free fluid in Douglas pouch and no adnexial pathology was observed. Based on these findings a definitive diagnosis of CSEP was made. The patient was informed of all possible treatment options and complications and adequately counseled. Because of the high hCG level, relatively advanced gestational age, and proximity of the gestational sac and the scar site from the previous cesarean section, medical management was rejected in favor of laparoscopic resection. During laparoscopy, the Fallopian tubes and both ovaries appeared normal, and no free peritoneal fluid or hematoma was observed. At first glance, no gestational sac protruding from the outer uterine surface was visible. Therefore, the vesicouterine peritoneum was incised to evaluate the scar site of the previous cesarean section(s). As the gestational sac was very close to the endometrial cavity, there was no visible swelling that corresponded to the gestational sac, and real time TVUS under direct laparoscopic supervision was used to confirm the location of the gestational sac. Subsequently, using an 8 mm Karman aspiration cannula, the gestational sac was evacuated by vacuum aspiration without any complications. A postprocedure TVUS showed a significant decrease in the size of the gestational sac and the patient was discharged without any complaints on postoperative day 2. The patient's hCG levels dropped to 901 mIU/mL after one week and were below 5 mIU/mL after two weeks of surgery.

## 3. Discussion

A cesarean scar ectopic pregnancy is a rare presentation and accounts for 6% of all ectopic pregnancies [1]. Its incidence is rapidly increasing over the years due to both a rise in cesarean rates and the use of improved diagnostic methods. Risk factors associated with a cesarean scar pregnancy are trauma to the myometrium caused by dilatation and curettage, prior cesarean section, myomectomy or an adenomyosis excision, pelvic inflammatory disease, the use of assisted reproductive techniques, and prior placental pathology [2, 3]. Most reported cases of CSEP appear to have been diagnosed in the first trimester [2, 4, 5].

Establishing a diagnosis of CSEP can be challenging and the preferred method of establishing a definitive diagnosis is TVUS with color, spectral, and power Doppler imaging. The sensitivity of the TVUS is quite satisfactory and has been reported to be 84.6% [6]. Another diagnostic tool used in CSEP is three-dimensional (3D) ultrasonography. It is being increasingly used as it allows surgeons to study a confined area in better detail [7]. In addition, magnetic resonance imaging and diagnostic laparoscopy may also be used to confirm the diagnosis. We detected the ectopic pregnancy in the anterior uterine wall by means of a real time 2D TVUS and Doppler imaging.

The most common differential diagnoses for CSEP are cervicoisthmic pregnancy and spontaneous abortion in progress. Several criteria for the diagnosis of CSEP by transvaginal sonography have been defined and a group of seven criteria proposed by Timor-Tritsch are as follows: (1) an empty uterine cavity and an empty endocervical canal, (2) a gestational sac located in the anterior portion of the lower uterine segment corresponding to the scar site of the previous cesarean, (3) demonstration of functional trophoblastic tissue by Doppler ultrasound at the site of implantation at the scar, (4) in early gestation, less than 8 weeks, a triangular shaped gestational sac filling the scar niche (after 8 weeks of gestation a rounded or an oval sac could be observed), (5) cervical canal that is closed and empty, (6) observation of fetal pole and/or yolk sac with or without heart activity, and (7) absence or deficiency of a healthy myometrium between the bladder and the gestational sac [8]. The last criterion allows differentiation of CSEP from a cervicoisthmic implantation [9].

Given the relatively rare incidence of CSEP, its management is controversial and current standards of therapy have been derived from data obtained from a limited number of patients. Management options include medical treatment with intralesional or systemic methotrexate and surgical intervention [10, 11].

Conservative management of CSEP carries a significant risk of bleeding and is generally not recommended. Systemic therapy with methotrexate is also not as effective for CSEP as it is for tubal ectopic pregnancies. However, an intralesional methotrexate injection, either through a transabdominal or a transvaginal route under US guidance, is quite successful, but normalization of hCG levels and shrinkage of the sac take longer with these treatment modalities [9].

Uterine artery embolization (UAE) is another option for nonsurgical treatment of CSEP. In recent studies UAE along with intra-arterial methotrexate injection was reported with high success rates. However, similar to intralesional methotrexate treatment, absorption of gestational sac and decline of hCG levels require relatively long time interval and significant bleeding could be observed in the follow-up period. Therefore suction curettage could be a safe option after UAE and methotrexate treatment in which vaginal bleeding persists [12].

Surgical intervention is another reliable treatment option for CSEP. Conventionally, a laparotomy and a resection of the ectopic sac along with the previous scar tissue have been used, but, in skilled hands, a laparoscopic excision alone is sufficient for complete treatment of CSEP. Further, patients presenting with an exogenously located CSEP are ideal candidates for laparoscopic intervention [7]. In a current literature review by Kanat-Pektas et al., 274 papers were evaluated with respect to different treatment modalities [13]. Methotrexate treatment was found to be the least effective method. Hysterotomy with either laparotomy or laparoscopy was quite successful with low failure rates. With respect to minimal invasive surgical approach, robotic assisted laparoscopic removal of residual cesarean ectopic pregnancy was also reported by Schmitt et al. In this report successful treatment of a persistent CSEP by robotic surgery after multiple intralesional methotrexate injections was described. Interestingly preoperative uterine artery embolization along with intraoperative temporary one sided uterine artery occlusion was performed [14].

Two types of CSEP have been defined based on the location of the gestational sac with respect to the uterine myometrial wall. In the first type (CSP-I), the conceptus is implanted in the previous scar and grows progressively into the cervicoisthmus space, while in the second type (CSP-II) the conceptus is implanted outside the myometrial scar and into the vesicouterine space [5]. Generally, blind curettage to evacuate a CSP-II is not recommended and is indeed dangerous as this could cause inadvertent perforation and profuse bleeding [15].

In our case, based on the findings from the TVUS, we initially planned to excise the gestational sac protruding from the outer surface of the uterus. Even though the sac appeared to be located through the uterine cavity, we thought that, after excision of bladder peritoneum, there would be a demarcation line that would clearly indicate the correct position of the gestational sac. However, during laparoscopy, it was found that the gestational sac was deeply implanted. Therefore, after confirming the location of the sac using a transvaginal US, we successfully vacuum aspirated the sac under direct laparoscopic guidance.

In conclusion, individualized treatment options based on gestational age, fetal viability, severity of symptoms, serum hCG levels, and ultrasonography findings are necessary for the successful treatment for CSEP. An early and timely diagnosis increases success rate and decreases complications to a considerable extent. The combined use of laparoscopic evaluation and ultrasound guidance for the aspiration in CSEP is an effective treatment strategy, particularly for ectopic sacs that are deeply implanted through the uterine wall.
